# Demonstration of Therapeutic Equivalence of Fluconazole Generic Products in the Neutropenic Mouse Model of Disseminated Candidiasis

**DOI:** 10.1371/journal.pone.0141872

**Published:** 2015-11-04

**Authors:** Javier M. Gonzalez, Carlos A. Rodriguez, Andres F. Zuluaga, Maria Agudelo, Omar Vesga

**Affiliations:** 1 GRIPE (Grupo Investigador de Problemas en Enfermedades Infecciosas), Universidad de Antioquia, Medellín, Colombia; 2 Scientific Direction, Clínica Cardio VID, Medellín, Colombia; 3 Infectious Diseases Unit, Hospital Universitario San Vicente Fundación, Medellín, Colombia; University of Wisconsin Medical School, UNITED STATES

## Abstract

Some generics of antibacterials fail therapeutic equivalence despite being pharmaceutical equivalents of their innovators, but data are scarce with antifungals. We used the neutropenic mice model of disseminated candidiasis to challenge the therapeutic equivalence of three generic products of fluconazole compared with the innovator in terms of concentration of the active pharmaceutical ingredient, analytical chemistry (liquid chromatography/mass spectrometry), *in vitro* susceptibility testing, single-dose serum pharmacokinetics in infected mice, and *in vivo* pharmacodynamics. Neutropenic, five week-old, murine pathogen free male mice of the strain Udea:ICR(CD-2) were injected in the tail vein with *Candida albicans* GRP-0144 (MIC = 0.25 mg/L) or *Candida albicans* CIB-19177 (MIC = 4 mg/L). Subcutaneous therapy with fluconazole (generics or innovator) and sterile saline (untreated controls) started 2 h after infection and ended 24 h later, with doses ranging from no effect to maximal effect (1 to 128 mg/kg per day) divided every 3 or 6 hours. The Hill’s model was fitted to the data by nonlinear regression, and results from each group compared by curve fitting analysis. All products were identical in terms of concentration, chromatographic and spectrographic profiles, MICs, mouse pharmacokinetics, and *in vivo* pharmacodynamic parameters. In conclusion, the generic products studied were pharmaceutically and therapeutically equivalent to the innovator of fluconazole.

## Introduction

Invasive candidiasis is rising in hospitalized patients, mainly in intensive care units [1], with an overall mortality comparable to that of severe sepsis [2]. Although new antifungal agents are available, fluconazole remains the most used agent for these infections in most settings [3–5]. Fluconazole is a purely synthetic bis-triazole derivative developed in early 1980s [6] and its patent expired several years ago allowing licensing of many generic products enormously cheaper than the innovator [7].

Data from animal models have demonstrated that generic products of many pharmaceutically equivalent antibiotics fail therapeutic equivalence when compared with the innovator [8–10]. In addition, therapeutic failure was shown for “bioequivalent” vancomycin in a case report [11] and for generic cefuroxime in a large clinical trial [12]. Of note, therapeutic equivalence was achievable for the intravenous forms of all generic products of two synthetic antibiotics: metronidazole [13] and ciprofloxacin [14]. Similarly, one generic product of Amphotericin B showed efficacy and safety similar to the innovator in the invasive pulmonary aspergillosis model in neutropenic rabbits [15], and one double-blind randomized trial demonstrated that the efficacy of generic products of itraconazole was not different from the innovator in the treatment of tinea pedis [16]. Regarding fluconazole, previous studies have found bioequivalence (i.e., PK equivalence) of oral generic formulations of fluconazole in healthy volunteers [17, 18], but there are no clinical or animal model studies with fluconazole generics in invasive candidiasis.

Based on this body of data, the determination of therapeutic equivalence of any generic antimicrobial cannot be assumed but requires *in vivo* experimentation in appropriate animal models; clinical trials have not only ethical barriers, but are prohibitively expensive having so many generic products in the market (for instance, 14 intravenous fluconazole products licensed by the Colombian drug regulatory agency by 2012). For this purpose, we compared with the innovator three generic products of parenteral fluconazole in terms of concentration of the active pharmaceutical ingredient, analytical chemistry, bioequivalence (mouse pharmacokinetics), *in vitro* susceptibility testing, and *in vivo* efficacy in the neutropenic mouse model of disseminated candidiasis.

Preliminary results of this work were presented at the 52^nd^ ICAAC [19].

## Materials and Methods

### Drugs

The innovator (Diflucan^®^, Pfizer PGM, France) and three generic products of fluconazole (FLC) marketed by Claris Pharmaceutical (Tergonil^®^, India), Fressenius-Kabi (Laboratorio Sanderson S.A., Chile) and Vitalis (Vitrofarma, Colombia), were bought as ready-to-use liquid solutions at local drugstores (Table 1). All products were licensed for human use by the drug regulatory agency of Colombia (INVIMA). The reference standard for analytical chemistry (fluconazole powder) was acquired from Sigma-Aldrich (Germany).

**Table 1:** Fluconazole products included in the study.

FLC Distributor	Pharmaceutical Form	Lot number	Local Price($USD)*
**Pfizer (innovator)**	Vial 200 mg/100 mL	A000704, A102704	75
**Claris Pharmaceutical**	Bag 200 mg/100 mL	A116897	3
**Fresenius-Kabi**	Bag 200 mg/100 mL	75EL2941	5
**Vitalis**	Vial 200 mg/100 mL	V111186	20

* The price was estimated with the exchange rate at the time of the study (2012).

### Organisms

We used two clinical isolates from patients with candidemia in all experiments: the wild-type strain *Candida albicans* GRP-0144 (FLC MIC 0.25 mg/L) and a borderline susceptible strain, *C*. *albicans* CIB-19177 (FLC MIC 4 mg/L). Other clinical isolates (*C*. *albicans* GRP-0143, *C*. *parapsilosis* GRP-0148 and *C*. *glabrata* GRP-0145) and the reference strain *C*. *albicans* ATCC 90028 (as MIC control organism) were included for *in vitro* susceptibility testing. For *in vivo* experimentation, the microorganisms were recovered from the ultrafreezer (-70°C), plated directly on Sabouraud dextrose agar (Difco Laboratories, USA), and incubated at 25°C for 30 h. This temperature was selected to favor the yeast over the filamentous form [20]. Prior to mouse inoculation, a few colonies were suspended in 5 mL of sterile saline to obtain a 530 nm optical density of 0.30 corresponding to ∼7 log_10_ CFU/mL.

### In vitro susceptibility testing

For susceptibility testing, we performed broth microdilution following CLSI protocol M27-A3 [21]. *Candida albicans* ATCC 90028 was the quality control organism. We run all assays by duplicate at least twice and recorded the geometric mean of the minimal inhibitory concentration (MIC) for each one of the study organisms.

### Pharmaceutical equivalence determined by LC/MS

We subjected the study products to liquid chromatography—mass spectrometry (LC/MS) to determine the concentration of the active pharmaceutical ingredient and the presence of contaminants. Analytical chemistry data were obtained with an Agilent 1100 liquid chromatograph coupled to a mass spectrometer electrospray ionization VL system. At the stationary phase, an analytical column of 150 mm by 4.6 mm of internal diameter (Hypersil Gold^™^ C18 selectivity column, Thermo Scientific) with 5 μm of particle size was employed per product. The single-ion monitoring (SIM) mode [M + H]^+^ was used to obtain the chromatogram, and the SCAN mode to gather the mass spectra, with a range of 100 to 1000 *m/z*. The mobile phase consisted of 10 mM ammonium acetate plus acetonitrile with 0.1% formic acid at 91:9 (volume) dilution. The pharmaceutical forms of fluconazole products were used for method development, and all preparations for reference material and pharmaceutical formulations were freshly prepared in deionized water for each analysis at fluconazole concentrations ranging from 0.2 to 2 mg/mL. The mobile phase was kept running in the equipment for 15 min prior to sampling; the sample volume was 10 μL and the run time lasted 5 min. Calibration curve experiments were performed by duplicate. Each quality control sample was analyzed three or four times in every assay. Between-run accuracy and precision were calculated for the calibration and quality control samples.

### The neutropenic mouse model of disseminated candidiasis

We used the neutropenic mouse model of disseminated candidiasis to test for bioequivalence (mouse pharmacokinetics) and to determine *in vivo* efficacy (pharmacodynamics) [22]. The animals were 5-week-old, 23–27 g, murine pathogen free, Swiss albino male mice of the strain Udea:ICR(CD-2) bred in our high-tech microisolation animal facility [23]. Immunosuppression was achieved with intraperitoneal injections of cyclophosphamide (Endoxan^®^, Baxter, Germany) administered 4 days (150 mg/kg) and 1 day (100 mg/kg) before infection; we demonstrated before that this protocol leads to profound neutropenia in our outbred mice (≤10 neutrophils/mm^3^) during at least 3 days counted after the second dose [24]. To induce fungemia, we placed the animals in a warming cage for a few minutes before injecting in the lateral tail vein 0.1 mL of a suspension of *Candida* blastospores containing ∼5.0 log_10_ CFU/mL. *In vivo* yeast burden was quantified by the number of colony forming units (CFU) in the kidneys 0, 2 and 26 h after infection. At these time-points, 3 mice were sacrificed by cervical dislocation under isoflurane sedation, and their kidneys were removed, homogenized (PRO200, ProSientific, USA), diluted for plating and incubated for 24 h at 37°C. To determine the lethality of the model in untreated animals, an additional group of 3 mice was followed for 120 h after infection, checking the animals every 24 h for survival. Animals were euthanized by cervical dislocation under isoflurane sedation when any of these criteria was fulfilled: (a) inability to obtain feed or water, or (b) no response to gentle stimuli or moribund state [25]. Animals were bred and maintained in the pathogen-free vivarium of the University of Antioquia; transfer to the experimental area occurred 24 h before starting immunosuppression. They were always fed and watered *ad libitum*, housed at a maximum density of 7 animals per box within a 693 cm^2^ area in a One Cage System^®^ (Lab Products, USA), and kept under controlled temperature between 22°C and 25°C. The study was reviewed and approved by the University of Antioquia Animal Experimentation Ethics Committee (session act No. 55, 2009) and followed the national guidelines for biomedical research (Resolution 008430 of 1993 by the Colombian Health Minister, articles 87 to 93).

### Single-dose serum pharmacokinetics in infected mice

To test for bioequivalence, we determined the pharmacokinetic profile of each product in the animal model. Two hours after infection with *C*. *albicans* GRP-0144, neutropenic mice received a single subcutaneous injection of fluconazole of 0.2 mL containing one of three dose levels: 1, 4 or 16 mg/kg. For each dose level, groups of 3 mice were terminally sampled by decapitation under isoflurane anesthesia at 1, 4, 8, 12 and 24 h after dosing, which requires 45 animals per product for a total of 180 mice. The blood was allowed to clot at 4°C before centrifugation at 5000 rpm for 5 min; then, the serum was removed and frozen at -70°C. Innovator and generic products were tested simultaneously and serum concentrations were determined by LC/MS. The parametric population software S-ADAPT-TRAN [26] was used to fit different models to the data (1 or 2 compartments, linear or Michaelis-Menten elimination). Selection of the best-performing model was based on the objective function (-2 log-likelihood), the observed vs. predicted plots, and the residual analysis. Between-subject variability was expressed as a percent coefficient of variation (%CV), and the residual error included both additive (SD_intercept_) and proportional (SD_slope_) terms.

### In vivo pharmacodynamics (PD)

To compare *in vivo* efficacy, the pharmacodynamic profile of innovator and generic fluconazole was determined. Subcutaneous fluconazole therapy started 2 h after infection allocating at random a different group of mice to each product or to sterile saline for untreated controls. For *C*. *albicans* GRP-0144, six doses (1, 2, 3, 4, 8 and 16 mg/kg per day divided q6h in a volume of 0.2 mL) were tested per product using 3 mice per dose level, requiring 18 mice per FLC product plus 9 untreated controls per experiment. To test the repeatability and the reliability of the animal model as a tool to determine the PD of generic antifungals [27], three experiments were made in different days: the first including only the innovator and one generic (Claris), the second with the innovator and two generics (Claris and Fresenius), and a third experiment involving all products. Against *C*. *albicans* CIB-19177, we tested seven doses of each FLC product (8, 16, 24, 32, 48, 64 and 128 mg/kg per day divided q3h) allocating 3 mice per dose level, requiring 21 mice per product plus 9 untreated controls for each experiment. Two different experiments were made in different days: one with the innovator and Claris, and the other including the innovator, Fresenius and Vitalis. For each experiment, we sacrificed 3 untreated controls one minute after intravenous inoculation (-2 h), at the onset (0 h), and at the end of therapy (24 h). After finishing treatment with the experimental arms, all animals were euthanized and both kidneys removed under aseptic technique, homogenized, serially diluted, plated by duplicate on Sabouraud agar, and incubated at 37°C under air atmosphere for 24 h. Data were registered as log_10_ CFU/g and the limit of detection was 2.0 log_10_ CFU/g.

### Statistical analysis

The minimal inhibitory concentration of all generic products was compared with the innovator by Kruskal-Wallis test (KW) using Prism 6.05.

Bioequivalence was assessed by the test/reference ratio of the natural logarithm of the area under the concentration-time curve (AUC) from the highest (16 mg/kg) and lowest (1 mg/kg) doses used. The products were considered bioequivalent if the 90% confidence interval of the difference in AUC was between 0.80 and 1.25. The SIM module of the ADAPT 5 [28] program was used to estimate the AUC of the different doses and the *f*AUC/MIC, with a 12% protein binding estimation from the literature [29].

The *in vivo* dose-response relationship was analyzed using Hill’s equation with four parameters fitted by least-squares nonlinear regression (SigmaPlot 12.3):

$$E=E_{0}-\left(\frac{E_{max}\times \;D^{N}}{ED_{50}^{N}+\;D^{N}}\right)$$ (1)

where *E* (effect) corresponds to the fungal load in the kidneys after 24 h of treatment, *E*_*0*_ represents the number of yeast cells in the untreated controls at 24 h, *E*_*max*_ is the maximum antifungal effect in log_10_ CFU/g, *D* is the fluconazole dose in mg/kg per day, *ED*_*50*_ is the dose needed to reach 50% of the *E*_*max*_, and *N* is Hill’s slope [9]. The goodness of fit was assessed by the adjusted coefficient of determination (AdjR^2^), the standard error of estimate (S_y|x_), and the fulfillment of the normality and homoscedasticity assumptions [30]. Any parameter with a variance inflation factor (VIF) <10 was considered free of collinearity. The primary pharmacodynamic parameters obtained for generic products and the innovator were compared by curve fitting analysis (CFA) using Prism 6.05. The reliability of the animal model was assessed by testing the interday repeatability of the innovator’s PD results [27]. Accepting a 5% chance for a type I error, the treatment of 18 animals per product to compare 3 generics versus the innovator confers 86% power to reject the null hypothesis, assuming that the magnitude of the difference in antifungal efficacy is ≥0.6 log_10_ CFU/g and the standard error of estimates is ≤0.5 log_10_ CFU/g (SigmaPlot 12.3).

## Results

### *In vitro* activity

Table 2 shows the MIC geometric means and ranges of all FLC products against six strains of *Candida* spp. There were no significant differences between products against any of strains.

**Table 2:** Geometric mean MICs (range) of fluconazole products against 6 *Candida* strains.

*Candida* strain	Fluconazole products				P-value (KW)
	Pfizer (innovator)	Claris	Fresenius	Vitalis	
***C*. *albicans* ATCC 90028**	0.59 (0.50–1.00)	0.71 (0.50–1.00)	0.71 (0.50–1.00)	0.63 (0.25–1.00)	0.94
***C*. *albicans* GRP-0144**	0.25 (0.25–0.25)	0.25 (0.25–0.25)	0.25 (0.25–0.25)	0.25 (0.25–0.25)	1.00
***C*. *albicans* CIB-19177**	5.66 (4.00–8.00)	5.66 (4.00–8.00)	8.00 (4.00–16.0)	6.73 (4.00–16.0)	0.85
***C*. *albicans GRP-0143***	0.42 (0.25–0.50)	0.35 (0.25–0.50)	0.30 (0.25–0.50)	0.30 (0.25–0.50)	0.70
***C*. *parapsilosis GRP-0148***	1.00 (1.00–1.00)	1.00 (1.00–1.00)	1.00 (1.00–1.00)	1.00 (1.00–1.00)	1.00
***C*. *glabrata GRP-0145***	16.0 (16.0–16.0)	19.0 (16.0–32.0)	26.9 (16.0–64.0)	16.0 (16.0–16.0)	0.99

MIC, minimal inhibitory concentration; KW, Kruskal-Wallis test. All assays performed by duplicate, at least twice.

### Analytical chemistry (LC/MS)

The method was linear with r^2^ > 0.993 for all calibration curves. Lower limit of quantification (calculated as signal/noise ratio greater than 3) was 5 ng.mL^-1^. Precision, expressed as %CV of the average for quality controls of 0.5, 2, and 6 μg.mL^-1^, was 9.2, 7.6, and 8.0, respectively. Inaccuracy averaged less than 10% for all controls. The chromatograms (SIM mode) of the reference, innovator, and three generics of FLC did not show differences in retention times, the peaks of the analyte, or other peaks that could reflect contaminants or impurities (S1 Fig).

### Single-dose serum PK in infected mice

The one-compartment model with linear elimination and first-order absorption described best the kinetics of innovator and generic fluconazole products. Table 3 includes the values of the population parameters clearance (*CL*), volume of distribution (*V*) and absorption rate constant (*K*_*a*_) with their respective between-subject variability and standard errors as well as the bioequivalence test at doses of 1 and 16 mg/kg. In both doses, the 90% confidence intervals for the ratio of the natural logarithm of the AUC for the generics (test) relative to the innovator (reference) ranged from 0.91 to 1.08, indicating that all three generics were bioequivalents of the innovator.

**Table 3:** Population pharmacokinetics in mice of three generic products and the innovator of fluconazole.

PK Parameter (Units)	Mean PK Parameter Value of Fluconazole Products (%CV)			
	Pfizer(Innovator)	Claris	Fresenius	Vitalis
***CL* (L/h)**	0.004 (13.4)	0.004 (14.1)	0.004 (12.0)	0.004 (10.9)
***V* (L)**	0.02 (10.9)	0.02 (3.7)	0.02 (11.6)	0.02 (4.80)
***K***_***a***_ **(h** ^**-1**^ **)**	2.14 (15.9)	2.46 (19.2)	2.95 (23.7)	4.28 (75.5)
**SD**_**intercept**_	0.20	0.19	0.25	0.23
**SD**_**slope**_	0.0009	0.0002	0.001	0.02
**T-R (90%CI at 1 mg/kg)**	Reference	0.95–0.97	0.92–0.95	0.97–1.01
**T-R (90%CI at 16 mg/kg)**	Reference	0.91–1.04	0.91–1.02	0.97–1.08

*CL*, clearance; *V*, volume of distribution; *K*_*a*_, absorption rate constant; SD_intercept_, standard deviation of the intercept; SD_slope_, standard deviation of the slope. Three dose levels were tested for the PK analysis (1, 4 and 16 mg/kg). T-R (90%CI): 90% confidence interval of the test-reference AUC (in natural logarithms). Groups of three mice were terminally sampled at 1, 4, 8, 12 and 24 h (15 animals per dose, 45 by product).

### In vivo pharmacodynamics

**Fig 1:**
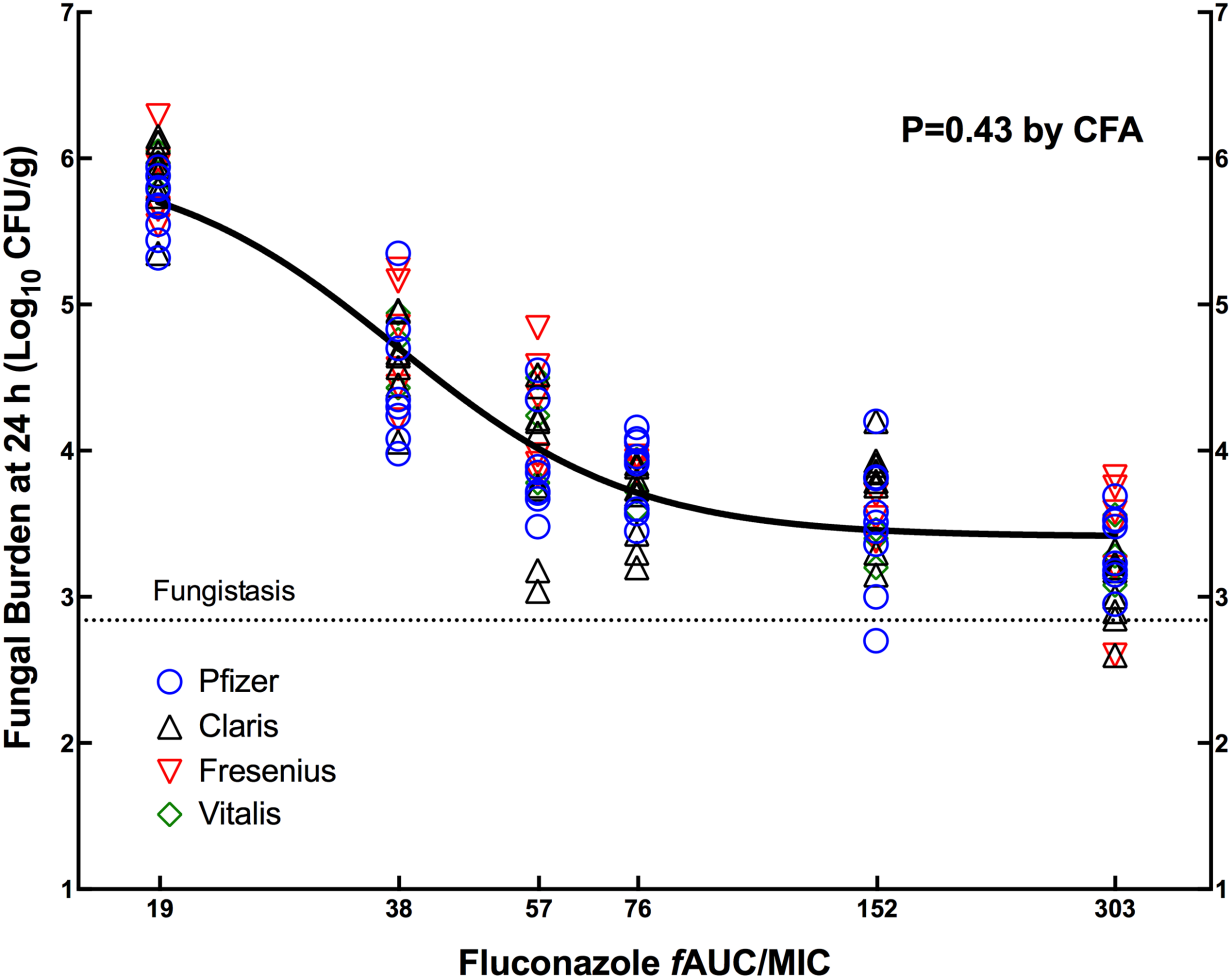
Pharmacodynamics of FLC generic products compared with the innovator against *C*. *albicans* GRP-0144. Data from three independent experiments were combined and analyzed by CFA. The innovator (Diflucan) and Claris products were included in all three experiments (54 animals per product), Fresenius in two (36 animals) and Vitalis in one (18 animals). A single curve (solid black line) described the data better than individual ones, indicating that the generics were therapeutically equivalent to the innovator. The horizontal dotted line indicates the fungal load at the beginning of therapy (0 h).

At the start of therapy, kidneys had 2.90 ± 0.24 and 2.94 ± 0.11 log_10_ CFU/g of *C*. *albicans* GRP-0144 and CIB-19177, respectively. The fungal burden over 24 h of *C*. *albicans* in the kidneys of untreated mice increased 2.41–3.37 and 3.06–3.08 log_10_ CFU/g for strains GRP-0144 and CIB-19177, respectively. The model lethality with both strains was 33% before 120 h. The repeatability of the PD results with the innovator against both strains of *C*. *albicans* confirmed the reliability of the animal model to study the PD of generic antifungals, i.e., in both cases the data obtained in different days were described by a single curve instead of individual ones (P of 0.1203 by CFA). Treatment with fluconazole produced no absolute reduction in colony counts compared with initial fungal burden, showing a fungistatic profile. Regarding therapeutic equivalence, Fig 1 illustrates the exposure-response curves for three generics and the innovator against *C*. *albicans* GRP-0144 in three independent experiments, and Table 4 shows the corresponding PD parameters. The regression of each product passed the normality and homoscedasticity tests, and the highest variance inflation factor for any parameter was 2.6, indicating the virtual absence of multicollinearity between the PD parameters. The CFA (P = 0.43) confirmed that the dose-effect data from the four products belonged to a single population, establishing the therapeutic equivalence of all three generics to the innovator. The *f*AUC/MIC corresponding to the *ED*_*50*_ was 37.9 ±1.4.

**Table 4:** Pharmacodynamic parameters of three generics and the innovator of FLC against *C*. *albicans* GRP-0144 (MIC = 0.25 mg/L) in the neutropenic mouse model of disseminated candidiasis.

PD Parameter (Units)	Mean PD Parameter Value of Fluconazole products (±SD)				
	Pfizer (innovator)	Claris	Fresenius	Vitalis	Global Curve*
***E***_***0***_ **(log**_**10**_ **CFU/g)**	5.95 (0.11)	5.98 (0.11)	6.07 (0.12)	6.04 (0.12)	6.00 (0.06)
***E***_***max***_ **(log**_**10**_ **CFU/g)**	2.52 (0.15)	2.57 (0.15)	2.63 (0.17)	2.72 (0.17)	2.59 (0.08)
***ED***_***50***_ **(mg/kg)**	1.88 (0.14)	1.97 (0.12)	2.12(0.16)	2.18 (0.15)	2.0 (0.07)
***f*AUC/MIC for *ED***_***50***_	35.5 (2.58)	37.5 (2.27)	40.1 (3.09)	41.3 (2.83)	37.9 (1.4)
***N* (Hill’s slope)**	2.78 (0.45)	3.56 (0.69)	2.56 (0.45)	2.90 (0.52)	2.93 (0.27)
**Adj.R** ^**2**^	0.89	0.89	0.91	0.96	0.90
**S**_**y|x**_ **(log**_**10**_ **CFU/g)**	0.33	0.36	0.30	0.22	0.33

*The P-value from the CFA was 0.43, indicating that a single global curve described all data. *E*_*max*_, maximum effect; *ED*_*50*,_ effective dose achieving 50% of the *E*_*max*_; *N*, slope; Adj.R^2^, adjusted coefficient of determination; S_y|x_, standard error of estimate; SE, standard error of the mean; CFA, curve-fitting analysis.

**Fig 2:**
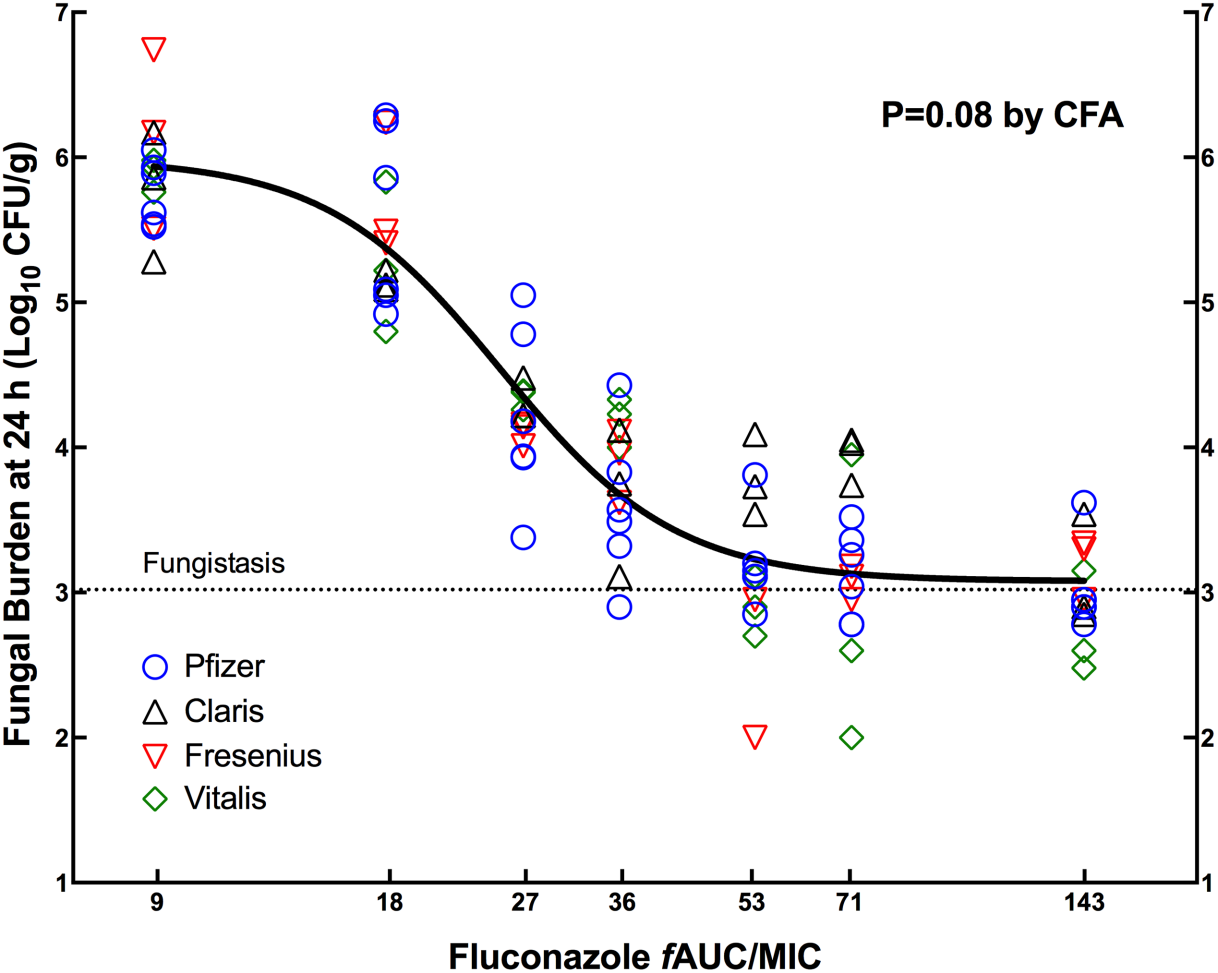
Pharmacodynamics of FLC generic products compared with the innovator against *C*. *albicans* CIB-19177. Data from two independent experiments were combined and analyzed by CFA. The innovator (Diflucan) was included in both experiments (42 animals), and Claris, Fresenius and Vitalis in one (21 animals per product). A single curve (solid black line) described the data better than individual ones, indicating that the generics were therapeutically equivalent to the innovator. The horizontal dotted line indicates the fungal load at the beginning of therapy (0 h).

Similar results were found against the less susceptible strain *C*. *albicans* CIB-19177. Fig 2 displays the exposure-response curves for the innovator and generic fluconazole products from two different experiments, and Table 5 shows the corresponding pharmacodynamic parameters. Again, the statistical comparison by CFA (P = 0.08) indicated that all data belonged to the same population and was best described by a single curve, confirming the therapeutic equivalence. Against this strain, the *f*AUC/MIC for *ED*_*50*_ was 25.1±1.03.

**Table 5:** Pharmacodynamic parameters of three generics and the innovator of FLC against *C*. *albicans* CIB-19177 (MIC = 4.0 mg/L) in the neutropenic mouse model of disseminated candidiasis.

PD Parameter(Units)	Mean PD Parameter Value of Fluconazole products (±SE)				
	Pfizer (innovator)	Claris	Fresenius	Vitalis	Global Curve*
***E***_***0***_ **(log**_**10**_ **CFU/g)**	5.93 (0.13)	6.02 (0.19)	6.12 (0.20)	6.00 (0.22)	5.99 (0.09)
***E***_***max***_ **(log**_**10**_ **CFU/g)**	2.83 (0.18)	2.55 (0.28)	3.09 (0.28)	3.40 (0.39)	2.91 (0.13)
***ED***_***50***_ **(mg/kg)**	22.6 (1.17)	18.9 (2.17)	22.5 (1.74)	26.2 (3.00)	22.5 (0.92)
***f*AUC/MIC for *ED***_***50***_	25.2 (1.31)	21.1 (2.43)	25.1 (1.95)	29.3 (3.35)	25.1 (1.03)
***N* (Hill’s slope)**	5.00 (1.13)	2.98 (0.95)	4.70 (1.48)	2.72 (0.80)	3.83 (0.55)
**Adj.R** ^**2**^	0.89	0.87	0.89	0.89	0.88
**S**_**y|x**_ **(log**_**10**_ **CFU/g)**	0.42	0.37	0.46	0.46	0.44

*The P-value from the CFA was 0.08, indicating that a single global curve described all data. *E*_*max*_, maximum effect; *ED*_*50*,_ effective dose achieving 50% of the *E*_*max*_; *N*, slope; Adj.R^2^, adjusted coefficient of determination; S_y|x_, standard error of estimate; SE, standard error of the mean; CFA, curve-fitting analysis.

## Discussion

Using the neutropenic model of disseminated candidiasis, we demonstrated that these three generic products of fluconazole were therapeutic equivalents of the innovator against two strains of *C*. *albicans* with a 16-fold difference in MIC. Considering that FLC prescriptions worldwide reach ∼60 defined daily doses (DDDs) per 1,000 patient days [4, 31, 32] demonstration of therapeutic equivalence of some generics is reassuring because they are ten times less expensive than the innovator, a price difference that represents huge savings for the health systems. For instance, 35.1 million patients are hospitalized every year in the United States with an average length of stay of 4.8 days, i.e., 168,480,000 patient-days per year [33]. The price of Diflucan^®^ is 60 USD per DDD (200 mg) [34], meaning that the total expenses of FLC in the US would ascend to 606.5 million dollars per year if the innovator still had patent exclusivity. In contrast, therapeutically equivalent generics would save 546 million dollars per year because their DDD costs only 6 USD.

We demonstrated before that therapeutic equivalence require absolute chemical identity of the active pharmaceutical ingredient [9, 10, 35]. Other key components of the pharmaceutical form must also demonstrate chemical identity in order to achieve therapeutic equivalence, as is the case of cilastatin in the imipenem formulation [36], or cyclodextrin in itraconazole [37] data also showed that, without exception, PK equivalence is certain as long as chemical identity is present (i.e.; pharmaceutical equivalence). However, and in spite of these findings, the only way to predict therapeutic equivalence is to demonstrate it in a suitable animal model of infection, because even apparently “identical” generics do fail in vivo [38–41]. Although it is obvious from this assertion that such “identity” cannot be present if a generic fails in vivo, the evidence shows that the chemical changes in the active pharmaceutical ingredient often appear after the drug is circulating in the living patient (animal or human), never before [10, 41]. These two examples illustrate the fact that the chemical identity goes quite far beyond the active pharmaceutical ingredient and the pharmaceutical form itself. True bioequivalence, as defined by identical efficacy in vivo (and not restricted to “similar” PK), requires the integration of PK and PD to label generic drugs as interchangeable.

In the case of FLC generics, their undistinguishable pharmacodynamic profiles are not surprising because it is a purely synthetic product that, when manufactured under a strict industrial process, delivers consistent quality [6]. It is designated as 2,4 (difluoro (bis (1H (1,2,4 (triazol (1 (ylmethyl) benzyl alcohol with an empirical formula of C_13_H_12_F_2_N_6_O and molecular weight 306.3. The commercial production process could generate intermediate and final compounds and an unwanted isomeric side product, but it can be removed easily from the mixture by methods such as chromatography on silica gel. The LC/MS data demonstrated the same concentration of the API in an identical state of purity, potency, contaminants, and degradation products, an important finding considering recent reports of impurities or contamination in generic products of heparins [42], methylprednisolone [43], and amphotericin B [44]. In contrast with antibiotics that are obtained by fermentation and purification processes (vancomycin, carbapenems or penicillin), synthetic antimicrobials like metronidazole [13], quinolones [14] and fluconazole are definitely easier to imitate. The generic products included in this study were manufactured in countries with different levels of industrialization (India, Chile and Colombia), demonstrating that a good manufacturing process is possible everywhere.

The mouse model of disseminated candidiasis simulates an invasive candidiasis, causing transitory fungemia and invasion of vital organs. It provides PK/PD parameters that have shown predictive validity in clinical studies [45] and can be compared statistically looking for differences between generic products of the same drug. In this case, the induction of neutropenia allows determination of the antimicrobial effect without the influence of the immune system. One limitation of this work is that we did not challenge FLC generics against species other than *Candida albicans* or fungal pathogens as important as *Cryptococcus neoformans*. In consequence, these findings cannot be extrapolated to such infections or to generic formulations of FLC not included in this study.

In conclusion, we have demonstrated the therapeutic equivalence of three generic products of fluconazole in the neutropenic mouse model of disseminated candidiasis. Patients, physicians, regulatory agencies and the pharmaceutical industry have now an experimental proof that these generics are indeed as effective *in vivo* as the innovator.

## Supporting Information

S1 Fig LC/MS analysis of the reference, innovator, and three generic products of fluconazole. The upper panel shows the chromatograms of the fluconazole products without differences in peaks and retention times; each color of the five curves represents a different product. The lower panel displays the centroid MS data of the five peaks, corresponding the molecular mass of fluconazole (307 Da).
(TIF)

S1 File *In vitro* activity of FLC generics against *Candida* spp. *In vitro susceptibility* data by broth microdilution of innovator and generic (Claris, Vitalis and Fresenius) FLC against *C*. *albicans* GRP-0144, *C*. *albicans* CIB-19177, *C*. *albicans* GRP-0143, *C*. *glabrata* GRP-0145 and *C*. *parapsilosis* GRP-0148. *Candida albicans* ATCC 90028 was used as the quality control organism. Data from four independent experiments are shown.
(XLSX)

S2 File First *in vivo* experiment against *C*. *albicans* GRP-0144, including Pfizer (innovator) and Claris FLC. *In vivo* pharmacodynamic data of innovator FLC and the generic Claris against *C*. *albicans* GRP-0144. The file contains the doses in mg/kg per day and the corresponding fungal burden at the end of treatment (in log_10_ CFU/g). The effect was calculated by subtracting the number of yeasts in treated animals from the untreated controls at 24h.
(XLS)

S3 File Second *in vivo* experiment against *C*. *albicans* GRP-0144, including Pfizer (innovator), Claris and Fresenius FLC. *In vivo* pharmacodynamic data of FLC generics (Claris and Fresenius) compared with the innovator (Pfizer) against *C*. *albicans* GRP-0144.
(XLS)

S4 File Third *in vivo* experiment against *C*. *albicans* GRP-0144, including Pfizer (innovator), Claris, Fresenius and Vitalis FLC. *In vivo* pharmacodynamic data of FLC generics (Claris, Fresenius and Vitalis) compared with the innovator (Pfizer) against *C*. *albicans* GRP-0144.
(XLS)

S5 File First *in vivo* experiment against *C*. *albicans* CIB-19177, including Pfizer (innovator) and Claris FLC. *In vivo* pharmacodynamic data of the FLC generic manufactured by Claris compared with the innovator against *C*. *albicans* CIB-19177. The file contains the doses in mg/kg per day and the corresponding fungal burden at the end of treatment (in log_10_ CFU/g). The effect was calculated by subtracting the number of yeasts in treated animals from the untreated controls at 24h.
(XLS)

S6 File Second *in vivo* experiment against *C*. *albicans* CIB-19177, including Pfizer (innovator), Fresenius and Vitalis FLC. *In vivo* pharmacodynamic data of the FLC generics manufactured by Fresenius and Vitalis compared with the innovator against *C*. *albicans* CIB-19177.
(XLS)

S7 File Pharmacokinetic data of Pfizer FLC. Data file of Pfizer fluconazole (innovator) for the pharmacokinetic analysis in S-ADAPT-TRAN.
(CSV)

S8 File Pharmacokinetic data of Claris FLC. Data file of Claris fluconazole (generic) for the pharmacokinetic analysis in S-ADAPT-TRAN.
(CSV)

S9 File Pharmacokinetic data of Fresenius FLC. Data file of Fresenius fluconazole (generic) for the pharmacokinetic analysis in S-ADAPT-TRAN.
(CSV)

S10 File PK data of Vitalis FLC. Data file of Vitalis fluconazole (generic) for the pharmacokinetic analysis in S-ADAPT-TRAN.
(CSV)

S11 File NC3Rs ARRIVE Guidelines. ARRIVE Guidelines Checklist file.
(PDF)
