# Supplementary figures and images for: Demonstration of Therapeutic Equivalence of Fluconazole Generic Products in the Neutropenic Mouse Model of Disseminated Candidiasis
Source: PLoS One. 2015 Nov 4;10(11):e0141872. doi: 10.1371/journal.pone.0141872 (PMC4633286; doi:10.1371/journal.pone.0141872)

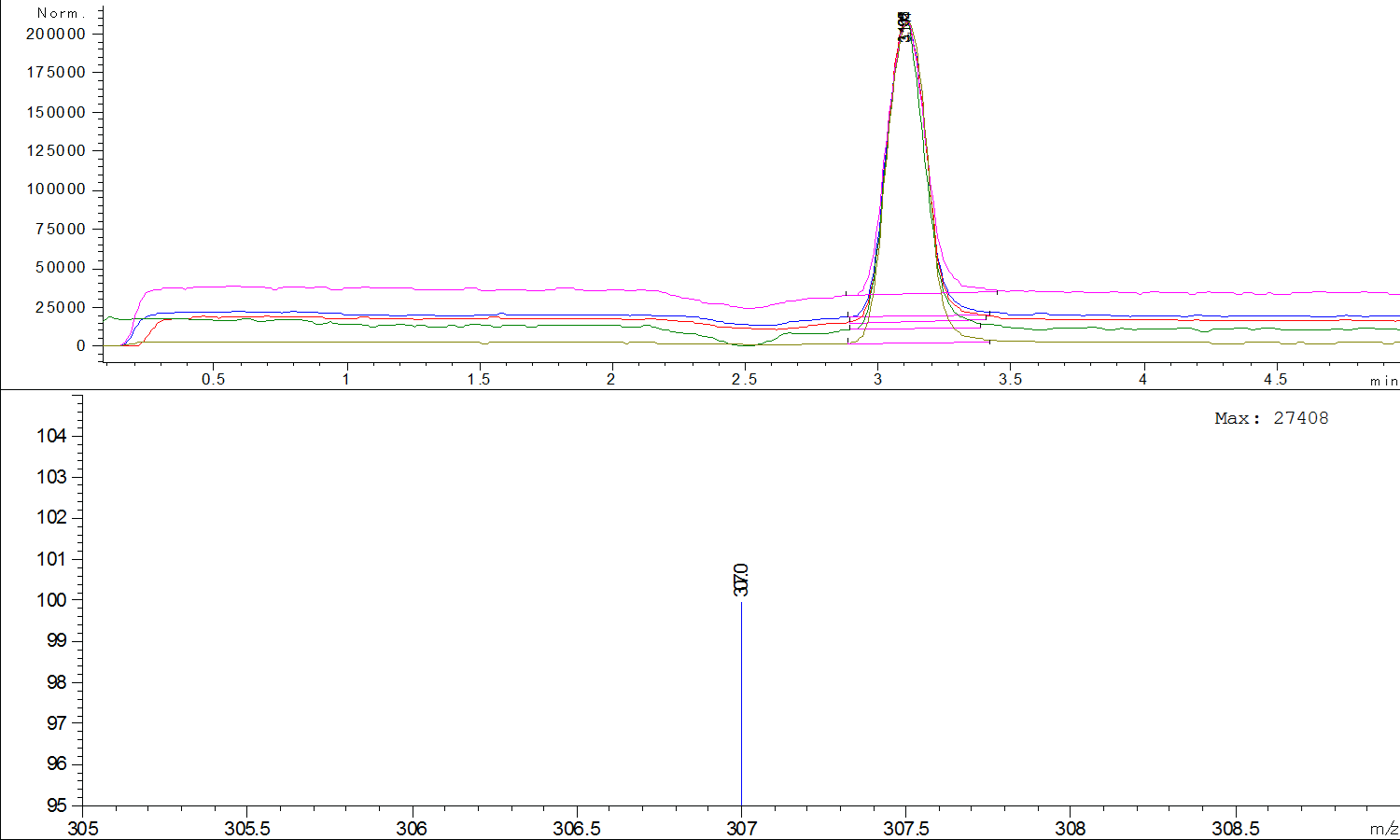

Supplement: S1 Fig — The upper panel shows the chromatograms of the fluconazole products without differences in peaks and retention times; each color of the five curves represents a different product. The lower panel displays the centroid MS data of the five peaks, corresponding the molecular mass of fluconazole (307 Da). (TIF) [file pone.0141872.s001.tif]
